# Letter to the Editor regarding: “Comparison between Fixation with Smooth Kirschner Wire and Cannulated Screws in Displaced Fractures of the Lateral Humeral Condyle in Children” – Vergara ADN, Fretes AN. Rev Bras Ortop 2023;58(1):149–156

**DOI:** 10.1055/s-0044-1779324

**Published:** 2024-04-24

**Authors:** Andreas Rehm, Ramy Shehata, Rachael Clegg, Sherif Elerian, Elizabeth Ashby

**Affiliations:** 1 Departamento de Ortopedia Pediátrica, Cambridge University Hospitals NHS Foundation Trust, Cambridge, Reino Unido

We read with interest the publication by Vergara and Fretes. 1 The authors only used the Weiss classification, 2 without measuring the amount of displacement for type III fractures (no data provided) and without recording if the fragment was rotated or not. All lateral humeral condyle fracture (LHCF) classifications have limitations, so that it is probably better to use 3 classifications, which complement each other by providing information on anatomy (Milch type 1 and 2), 3 displacement and malrotation of the fragment (Jacob 1 to 3) 4 and displacement and outcome (Weiss. I: <2mm; II:≥2- < 4mm with intact articular cartilage; III:≥4mm), 2 in addition to measuring the amount of displacement.

Bland et al. 5 reported that the internal oblique radiograph (IOR) is the most reliable view to measure displacement of LHCFs. Vergara and Fretes 1 did not take IORs, raising the possibility that some of the fracture classed as Weiss type II might have been a type III.

Weiss et al. 2 performed arthrograms on all patients with 2 to <4 mm displacement, which showed that all fractures had an intact articular cartilage and were therefore treated with closed reduction and percutaneous pinning (CRPP). We would like to ask Vergara and Fretes why they performed an open reduction in 4 of their Weiss type II fractures, which could have possibly been treated with CRPP instead?

Xie et al. 6 managed to perform CRPP in 74% of their Weiss type III fractures, with the success rate of performing a closed reduction having depended on the fracture anatomy. The success rate was 82% for Milch type 2 (fracture line runs through the trochlea) and 50% for type 1 fractures (line runs through the capitello-trochlear sulcus or lateral to it), without there having been a significant difference between displaced fractures with (Jakob type 3) and without fragment rotation.

Li et al. 7 performed ultrasounds and identified that the articular cartilage was intact in 14 of 39 children with Weiss type II fractures. Based on the latter Li et al 7 expanded the use of non-operative management to these 14 patients, which did not show further displacement in the casts.

Bernthal et al. 8 reported on 141 LHCFs, 76 treated non-operatively, 14 with CRPP and 51 with open reduction and percutaneous pinning (ORPP). Those treated with CRPP or ORPP had a significantly reduced absolute arc of motion up to 18 weeks after injury compared to the non-operative group, with 6 major complications in the ORPP group (4x osteonecrosis of the capitellum; 1x osteonecrosis of the trochlea; 1x deep infection), but none in the other two groups.

Nazareth et al. 9 reported significantly reduced PODCI scores for type II and III fractures at 6 and 12 weeks and no significant difference at 1 year, compared to normative data.

The above publications 8 9 show that patients who had non-operative management recover elbow function quicker and that ORPP is associated with a by far high major complication rate compared to non-operative management and CRPP. Vergara and Fretes' two cases with a major operative complication (1x radial nerve palsy and non-union; 1x non-union) had undergone an open reduction and screw fixation. This gives a major complication rate of 40% for this combination and 18% for screw fixation, which is by far higher than the 12% reported by Bernthal et al. 8 for ORPP. Vergara and Fretes presented radiographs of two patients who had undergone screw fixation. These show that both fixations are inadequate because of the screws not being perpendicular to the fracture line, reducing biomechanical stability by increasing shear forces across the fracture site, 10 thereby increasing failure risk. In contrary to the authors' claim that their study does not show any benefits in relation to the use of smooth pins or cannulated screws, we identified that there is a high major complication rate for screw fixations and surgeons seem to struggle to obtain a good screw position in relation to the fracture configuration.

There are inconsistencies in Vergara and Fretes' paper. 1 The authors first gave the impression as if they had conducted a prospective randomized trial:” The treatment was selected by drawing lots. The envelopes were selected in the preoperative holding area by the circulating nurse…” The authors then stated:” The decision on which implant to use was always made in the operating room, based on previous operative planning.” The latter statement contradicts the former. The authors stated that there was no difference regarding the observed range of movement between the two implant types at 12 weeks, not providing any measurements and not providing data on how long it took for the function to return to normal, since we know from the literature that it takes much longer than 12 weeks for function to recover. 8 9

In conclusion, Vergara and Fretes' data show a high major complication rate for open reductions and screw fixations. To use exuberant callus formation as an outcome measure to compare surgical techniques since is not useful since it is very common (73% of fractures). 11 The authors missed the opportunity to assess the intactness of the articular cartilage by performing arthrograms or ultrasounds, which could possibly help to increase the number of patients treated non-operatively and treated by CRPP, with non-operative treatment being associated with improved restoration of elbow function and non-operative management and CRPP potentially avoiding major complications associated with open reductions.

## References

[1] Vergara A DN Fretes A N Comparison between Fixation with Smooth Kirschner Wire and Cannulated Screws in Displaced Fractures of the Lateral Humeral Condyle in Children Rev Bras Ortop 2022 58 01 149 156 10.1055/s-0042-1757307 PMC10038712 36969771

[2] Weiss J M Graves S Yang S Mendelsohn E Kay R M Skaggs D L A new classification system predictive of complications in surgically treated pediatric humeral lateral condyle fractures J Pediatr Orthop 2009 29 06 602 605 19700990 10.1097/BPO.0b013e3181b2842c

[3] Milch H Fractures of the external humeral condyle J Am Med Assoc 1956 160 08 641 646 13286109 10.1001/jama.1956.02960430031006

[4] Jakob R Fowles J V Rang M Kassab M T Observations concerning fractures of the lateral humeral condyle in children J Bone Joint Surg Br 1975 57 04 430 436 1104630

[5] Bland D C Pennock A T Upasani V V Edmonds E W Measurement reliability in pediatric lateral condyle fractures of the humeus J Pediatr Orthop 2018 38 08 e429 e433 29917010 10.1097/BPO.0000000000001200

[6] Xie L W Tan G Deng Z Q Impacts of Fracture Types on Success Rate of Closed Reduction and Percutaneous Pinning in Pediatric Lateral Condyle Humerus Fractures Displaced >4 mm J Pediatr Orthop 2022 42 05 265 272 35180724 10.1097/BPO.0000000000002093

[7] Li X T Shen X T Wu X Chen X L A novel transverse ultrasonography technique for minimally displaced lateral humeral condyle fractures in children Orthop Traumatol Surg Res 2019 105 03 557 562 30935813 10.1016/j.otsr.2019.02.005

[8] Bernthal N M Hoshino C M Dichter D Wong M Silva M Recovery of elbow motion following pediatric lateral condylar fractures of the humerus J Bone Joint Surg Am 2011 93 09 871 877 21543677 10.2106/JBJS.J.00935

[9] Nazareth A VandenBerg C D Sarkisova N Prospective Evaluation of a Treatment Protocol Based on Fracture Displacement for Pediatric Lateral Condyle Humerus Fractures: A Preliminary Study J Pediatr Orthop 2020 40 07 e541 e546 31834242 10.1097/BPO.0000000000001491

[10] Schlitz R S Schwertz J M Eberhardt A W Gilbert S R Biomechanical Analysis of Screws Versus K-Wires for Lateral Humeral Condyle Fractures J Pediatr Orthop 2015 35 08 e93 e97 25985374 10.1097/BPO.0000000000000450

[11] Pribaz J R Bernthal N M Wong T C Silva M Lateral spurring (overgrowth) after pediatric lateral condyle fractures J Pediatr Orthop 2012 32 05 456 460 22706459 10.1097/BPO.0b013e318259ff63

