# Supplementary material for: Letter to the Editor regarding: “Comparison between Fixation with Smooth Kirschner Wire and Cannulated Screws in Displaced Fractures of the Lateral Humeral Condyle in Children” – Vergara ADN, Fretes AN. Rev Bras Ortop 2023;58(1):149–156
Source: Rev Bras Ortop (Sao Paulo). 2024 Apr 24;59(6):e1008–9. doi: 10.1055/s-0044-1779324 (PMC11663073; doi:10.1055/s-0044-1779324)
Supplement: Related texts

# Research article: Carta ao Editor sobre: “Comparação entre fixação de fio de Kirschner liso e de parafusos canulados em fraturas deslocadas do côndilo lateral do úmero em crianças” – Vergara ADN, Fretes AN. Rev Bras Ortop 2023;58(1):149–156

Lemos com interesse a publicação de Vergara e Fretes. 1 Os autores utilizaram apenas a classificação de Weiss, 2 sem medir a quantidade de deslocamento para as fraturas do tipo III (nenhum dado fornecido) e sem registrar se o fragmento estava rotacionado ou não. Todas as classificações de fratura do côndilo lateral do úmero (FCLU) têm limitações, sendo provavelmente melhor utilizar três classificações que se complementam, fornecendo informações sobre a anatomia (Milch tipo 1 e 2), 3 deslocamento e rotação do fragmento (Jacob 1 a 3), 4 e deslocamento e resultado (Weiss. I: <2 mm; II: ≥2- < 4 mm com cartilagem articular intacta; III: ≥4 mm), 2 além de medir a quantidade de deslocamento.

Bland et al. 5 relatou que a radiografia oblíqua interna (ROI) é a visão mais confiável para medir o deslocamento FCLU. Vergara e Fretes 1 não realizaram ROIs, levantando a possibilidade de que algumas das fraturas classificadas como Weiss tipo II poderiam ter sido do tipo III.

Weiss et al. 2 realizaram artrografias em todos os pacientes com deslocamento de 2 a <4 mm, o que mostrou que todas as fraturas tinham cartilagem articular intacta e, portanto, foram tratadas com redução fechada e fixação percutânea (RFFP). Gostaríamos de perguntar a Vergara e Fretes por que eles realizaram uma redução aberta em 4 de suas fraturas Weiss tipo II, que poderiam ter sido possivelmente tratadas com RFFP?

Xie et al. 6 conseguiram realizar RFFP em 74% de suas fraturas Weiss tipo III, com a taxa de sucesso da redução fechada dependendo da anatomia da fratura. A taxa de sucesso foi de 82% para o tipo 2 de Milch (a linha da fratura passa pela tróclea) e 50% para as fraturas do tipo 1 (a linha passa pelo sulco capitelo-tróclea ou lateral a ele), sem haver diferença significativa entre fraturas deslocadas com (tipo 3 de Jakob) e sem rotação do fragmento.

Li et al. 7 realizaram ultrassons e identificaram que a cartilagem articular estava intacta em 14 de 39 crianças com fraturas tipo II de Weiss. Com base nisso, Li et al 7 expandiram o uso do tratamento não operatório para esses 14 pacientes, que não apresentaram mais deslocamento nos gessos.

Bernthal et al. 8 relataram 141 fraturas do côndilo lateral do úmero, sendo 76 tratadas não cirurgicamente, 14 com RFFP e 51 com redução aberta e fixação percutânea (RAFP). Aqueles tratados com RFFP ou RAFP tiveram uma redução significativa no arco absoluto de movimento até 18 semanas após a lesão, em comparação com o grupo não operatório, com 6 complicações graves no grupo RAFP (4x osteonecrose do capitelo; 1x osteonecrose da tróclea; 1x infecção profunda), mas nenhuma nos outros dois grupos.

Nazareth et al. 9 relataram pontuações PODCI significativamente reduzidas para fraturas tipo II e III em 6 e 12 semanas e nenhuma diferença significativa em 1 ano, em comparação com dados normativos.

Os artigos mencionados 8 9 mostram que os pacientes que tiveram tratamento não operatório recuperam a função do cotovelo mais rapidamente e que a RAFP está associada a uma taxa de complicações maiores muito alta em comparação com o tratamento não operatório e a RFFP. Os dois casos de Vergara e Fretes com complicação operatória importante (1x paralisia e não consolidação do nervo radial; 1x não união) foram submetidos a redução aberta e fixação com parafuso. Isto dá uma taxa de complicações importantes de 40% para esta combinação e 18% para fixação com parafuso, o que é de longe superior aos 12% relatados por Bernthal et al. 8 para RAFP. Vergara e Fretes apresentaram radiografias de dois pacientes submetidos à fixação com parafusos. Estes mostram que ambas as fixações são inadequadas porque os parafusos não são perpendiculares à linha de fratura, reduzindo a estabilidade biomecânica ao aumentar as forças de cisalhamento no local da fratura, 10 aumentando assim o risco de falha. Contrariando a afirmação dos autores de que seu estudo não mostra nenhum benefício em relação ao uso de pinos lisos ou parafusos canulados, identificamos que há uma alta taxa de complicações maiores para fixações com parafusos e os cirurgiões parecem ter dificuldade para obter um bom parafuso posição em relação à configuração da fratura.

Existem inconsistências no artigo de Vergara e Fretes. 1 Os autores deram a impressão inicial de que conduziram um ensaio clínico randomizado prospectivo: "O tratamento foi selecionado por sorteio. Os envelopes foram selecionados na sala pré-operatória pela enfermeira circulante..." Os autores então afirmaram: "A decisão sobre qual implante usar sempre foi tomada na sala de cirurgia, com base em um planejamento operacional prévio." Essa última declaração contradiz a primeira. Os autores afirmaram que não houve diferença em relação à amplitude de movimento observada entre os dois tipos de implantes às 12 semanas, sem fornecer medições e sem apresentar dados sobre quanto tempo levou para que a função retornasse ao normal, uma vez que sabemos pela literatura que leva muito mais tempo do que 12 semanas para a função se recuperar. 8 9

Em conclusão, os dados apresentados por Vergara e Fretes mostram uma alta taxa de complicações significativas para reduções abertas e fixações com parafusos. Utilizar a formação de calo exuberante como medida de desfecho para comparar técnicas cirúrgicas não é útil, pois é bastante comum (73% das fraturas). 11 Os autores perderam a oportunidade de avaliar a integridade da cartilagem articular por meio de artrografias ou ultrassonografias, o que poderia possivelmente ter contribuído para aumentar o número de pacientes tratados de forma não cirúrgica, optando por RFFP. O tratamento não operatório está associado a uma melhor restauração da função do cotovelo, enquanto o manejo não operatório e a RFFP podem potencialmente evitar complicações significativas associadas às reduções abertas.
